# Decreased circulating dihomo-gamma-linolenic acid levels are associated with total mortality in patients with acute cardiovascular disease and acute decompensated heart failure

**DOI:** 10.1186/s12944-017-0542-2

**Published:** 2017-08-14

**Authors:** Shohei Ouchi, Tetsuro Miyazaki, Kazunori Shimada, Yurina Sugita, Megumi Shimizu, Azusa Murata, Takao Kato, Tatsuro Aikawa, Shoko Suda, Tomoyuki Shiozawa, Masaru Hiki, Shuhei Takahashi, Takatoshi Kasai, Katsumi Miyauchi, Hiroyuki Daida

**Affiliations:** Department of Cardiovascular Medicine, Juntendo University School of Medicine, 2-1-1 Hongo Bunkyo-ku, Tokyo, 113-8421 Japan

**Keywords:** Polyunsaturated fatty acids, Dihomo-gamma-linolenic acid, Arachidonic acid, Omega-6, Inflammation, Nutrition

## Abstract

**Background:**

Polyunsaturated fatty acids (PUFAs) have important roles in the pathogenesis of cardiovascular diseases. However, the clinical significance of omega-6 PUFAs in acute cardiovascular disease remains unknown.

**Methods:**

We enrolled 417 consecutive patients with acute cardiovascular disease admitted to the cardiac intensive care unit at Juntendo University Hospital between April 2012 and October 2013. We investigated the association between serum PUFA levels and long-term mortality. Blood samples were collected after an overnight fast, within 24 h of admission. We excluded patients who received eicosapentaenoic acid therapy and those with malignancy, end-stage kidney disease, chronic hepatic disease, and connective tissue disease.

**Results:**

Overall, 306 patients (mean age: 66.4 ± 15.0 years) were analysed. During the follow-up period of 2.4 ± 1.2 years, 50 patients (16.3%) died. The dihomo-gamma-linolenic acid (DGLA) levels, arachidonic acid (AA) levels, and DGLA/AA ratio were significantly lower in the nonsurvivor group than in the survivor group (DGLA: 23.2 ± 9.8 vs. 31.5 ± 12.0 μg/ml, AA: 151.1 ± 41.6 vs. 173.3 ± 51.6 μg/ml, and DGLA/AA: 0.16 ± 0.05 vs. 0.19 ± 0.06, all *p* < 0.01). Kaplan–Meier curves showed that survival rates were significantly higher in the higher DGLA, AA, and DGLA/AA groups than in their lower counterparts (DGLA and AA; *p* < 0.01, DGLA/AA; *p* = 0.01), although omega-3 PUFAs were not associated with prognosis. Furthermore, in patients with acute decompensated heart failure (ADHF), survival rates were significantly higher in the higher DGLA, AA, and DGLA/AA groups than in their lower counterparts (DGLA and AA; *p* < 0.01, DGLA/AA; *p* = 0.04). However, among patients with acute coronary syndrome, none of the PUFA levels were associated with prognosis. Among patients with ADHF, after controlling for confounding variables, DGLA and DGLA/AA were associated with long-term mortality [DGLA: hazard ratio (HR), 0.94; 95% confidence interval (CI), 0.88–0.99; *p* = 0.01 and DGLA/AA: HR, 0.87; 95% CI, 0.77–0.97; *p* < 0.01], whereas AA was not associated with prognosis.

**Conclusion:**

Low omega-6 PUFA levels, particularly DGLA, and a low DGLA/AA ratio predict long-term mortality in patients with acute cardiovascular disease and ADHF.

**Trial registration:**

UMIN-CTR; UMIN000007555.

**Electronic supplementary material:**

The online version of this article (doi:10.1186/s12944-017-0542-2) contains supplementary material, which is available to authorized users.

## Background

Polyunsaturated fatty acids (PUFAs) play structural and functional roles as membrane components and precursors for physiologically active substances involved in inflammation [1]. PUFAs are characterized by the presence of at least two carbon-carbon double bonds. Omega-3 PUFAs [eicosapentaenoic acid (EPA) and docosahexaenoic acid (DHA)] have the first double bond at the third carbon from the methyl terminus, and omega-6 PUFAs [arachidonic acid (AA) and dihomo-gamma-linolenic acid (DGLA)] have the first double bond at the sixth carbon from the methyl terminus [2, 3]. Fish oils are rich in omega-3 PUFAs, whereas farm animals and sunflower, safflower, and corn oils are rich in omega-6 PUFAs [4, 5]. A previous study reported that in Japanese individuals, the levels of EPA, DHA, EPA/AA, and other omega-3/omega-6 PUFAs in serum and red blood cells gradually increased with age, owing to the westernization of lifestyles in young people including the increased availability of high-fat foods [6].

PUFAs, particularly omega-3 PUFAs, have several important roles in the prevention of cardiovascular diseases and have anti-inflammatory and anti-atherogenic effects [7–14]. Previous studies have reported that treatment with omega-3 PUFAs increases the left ventricular ejection fraction in patients with chronic heart failure [5, 15–17]. However, it is unclear whether omega-6 PUFAs are beneficial or harmful with regard to the prevention of cardiovascular diseases [18–22]. Moreover, the clinical significance of PUFA metabolism, including that of omega-6 PUFAs, in acute cardiovascular diseases remains unknown. Therefore, we investigated the association between serum PUFA levels and prognosis in patients with acute cardiovascular disease.

## Methods

### Patients

**Fig. 1:**
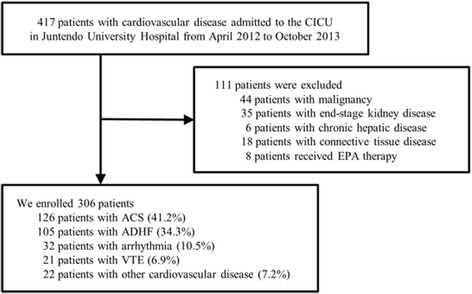
Flowchart of the study population

The present study was a part of an ongoing cohort study of biomarkers in patients admitted to the cardiac intensive care unit (UMIN-CTR; UMIN000007555), in which the PUFA hypothesis was generated retrospectively; however, data were collected systematically and prospectively. We enrolled 417 consecutive patients with cardiovascular diseases admitted to the cardiac intensive care unit at Juntendo University Hospital between April 2012 and October 2013. We excluded patients who received EPA therapy and those with malignancy, end-stage kidney disease [defined as an estimated glomerular filtration rate (eGFR) <15 ml/min/1.73 m^2^], chronic hepatic disease, and connective tissue disease (Fig. 1). The primary endpoint was all-cause death, and we followed up with the patients until December 2015.

Overall, 306 patients were analysed in this study. The mean patient age was 66.4 ± 15.0 years. Of the 306 patients, 209 (68.3%) were male. All patients were Japanese. Juntendo University Hospital is located in Tokyo, the capital of Japan, and none of the patients were employed in a primary industry. Based on socio-economic status, the patients included 145 retirees or housewives (47.4%), 130 white-collar workers (42.5%), 6 physical laborers (2.0%), and 5 who received public assistance (1.6%). The occupations of the other 20 patients (6.5%) were unclear from the medical records. The mean follow-up duration was 2.4 ± 1.2 years, and the maximum follow-up duration was 3.7 years.

Acute decompensated heart failure (ADHF) was defined according to the diagnostic criteria of the Framingham study [23]. Acute coronary syndrome (ACS) was defined as having an unstable angina pectoris, a non-ST elevation, or a ST elevation myocardial infarction. Diabetes mellitus was defined as having a previous diagnosis of diabetes mellitus in the medical records, a hemoglobin A1c (national glycohemoglobin standardization program calculation) level ≥ 6.5%, or treatment with oral antidiabetic agents or insulin. Dyslipidemia was defined as having a previous diagnosis of dyslipidemia in the medical records, abnormal lipid profiles [i.e., triglyceride (TG) level ≥ 150 mg/dl, low-density lipoprotein cholesterol (LDL-C) level ≥ 140 mg/dl, or high-density lipoprotein cholesterol (HDL-C) level ≤ 40 mg/dl], or treatment with antidyslipidemic agents. Hypertension was defined as having a previous diagnosis of hypertension, which was defined as having a systolic blood pressure of ≥140 mmHg and/or diastolic blood pressure of ≥90 mmHg [24] in the medical records or treatment with antihypertensive agents. All subjects provided informed consent, and the study was approved by the Ethical Committee of Juntendo University Hospital.

### Blood sampling

Whole blood samples were collected after an overnight fast, within 24 h of admission, and were analysed using standardized methods. Tubes for measuring PUFAs contained heparin sodium, those for measuring HbA1c levels contained heparin sodium, ethylenediaminetetraacetic acid disodium salt and sodium fluoride, those for measuring brain natriuretic peptide (BNP) levels contained ethylenediaminetetraacetic acid dipotassium salt, and those for measuring total cholesterol, TG, HDL-C, creatinine, total protein, and albumin contained a fast-acting coagulant. The LDL-C level was calculated using the Friedewald formula. eGFR was calculated based on the Japanese equation that uses serum creatinine level, age, and gender as follows: eGFR (mL/min/1.73 m^2^) = 194 × creatinine^−1.094^ × age^−0.287^ (for females = × 0.739) [25]. Serum concentrations of EPA, DHA, DGLA, and AA were measured using standard laboratory protocols by SRL Inc. (Tokyo, Japan).

### Statistical analyses

Continuous variables were expressed as means with standard deviations, and categorical variables were expressed as counts and percentages. Comparisons of continuous variables were performed using the Student’s *t*-test or Mann–Whitney *U*-test. Categorical variables were analysed using the chi-square test or Fisher’s exact probability test. Correlations between two variables were determined using simple linear regression analysis. The patients were divided into survivor or nonsurvivor groups based on their survival. Unadjusted cumulative event rates for the primary endpoint were estimated using the Kaplan–Meier method and were compared between groups using the log-rank test. We defined cut-offs using the median PUFA levels. We analysed the long-term mortality of patients with ADHF and those with ACS. Among patients with ADHF, univariate and multivariate Cox regression analyses were performed to identify predictors of the primary endpoint. The hazard ratios (HRs) and 95% confidence intervals (CIs) were also calculated. Age, sex, body mass index (BMI), diabetes mellitus, dyslipidemia, hypertension, renal function (eGFR), atrial fibrillation, and cardiac function (left ventricular ejection fraction) were included in the multivariate analyses. JMP12 (for Windows, SAS Institute, Cary, NC) was used for the statistical analysis, and *p-*values <0.05 were considered statistically significant.

## Results

The survivor group included 256 patients (83.7%), and the nonsurvivor group included 50 patients (16.3%). Age and prevalence of atrial fibrillation were significantly higher and BMI, systolic blood pressure, diastolic blood pressure, ejection fraction, and prevalence of dyslipidemia were significantly lower in the nonsurvivor group than in the survivor group. Total cholesterol, LDL-C, TG, and albumin levels were significantly lower, BNP levels were significantly higher, and impaired renal function was more common in the nonsurvivor group than in the survivor group. The administration of diuretics, anticoagulants, and inotropic agents was more common in the nonsurvivor group than in the survivor group (Table 1).

**Table 1:** Characteristics of the study subjects

	Survivor group (*n* = 256)	Nonsurvivor group (*n* = 50)	*p*
Age (years)	64.7 ± 15.2	75.3 ± 10.7	<0.01
Male (n, %)	175 (68.4)	34 (68.0)	NS
Body mass index (kg/m^2^)	24.2 ± 4.5	22.1 ± 3.8	<0.01
Systolic blood pressure (mmHg)	134 ± 27	124 ± 32	0.03
Diastolic blood pressure (mmHg)	78 ± 19	70 ± 18	<0.01
Heart rate (/min)	85 ± 30	82 ± 25	NS
Ejection fraction (%)	51.6 ± 16.7	39.7 ± 20.1	<0.01
Diabetes mellitus (n, %)	86 (33.6)	21 (42.0)	NS
Dyslipidemia (n, %)	191 (74.6)	28 (56.0)	0.01
Hypertension (n, %)	174 (68.0)	34 (68.0%)	NS
Atrial fibrillation (n, %)	59 (23.1)	22 (44.0)	<0.01
Ischemic heart disease (n, %)	141 (55.1)	27 (54.0)	NS
Diagnosis			
Acute decompensated heart failure (n, %)	71 (27.7)	34 (68.0)	<0.01
Acute coronary syndrome (n, %)	121 (47.3)	8 (16.0)	<0.01
Laboratory data			
Total cholesterol (mg/dL)	172 ± 38	153 ± 27	<0.01
LDL-cholesterol (mg/dL)	105 ± 31	93 ± 21	0.03
HDL- cholesterol (mg/dL)	44 ± 14	42 ± 12	NS
Triglycerides (mg/dL)	116 ± 72	86 ± 39	0.01
eGFR (mL/min/1.73m^2^)	71.9 ± 27.4	45.4 ± 26.8	<0.01
Hemoglobin A1c (%)	6.2 ± 1.2	6.1 ± 0.8	NS
Total protein (g/dL)	6.6 ± 0.6	6.5 ± 0.6	NS
Albumin (g/dL)	3.8 ± 0.6	3.4 ± 0.5	<0.01
Brain natriuretic peptide (pg/mL)	490 ± 762	1443 ± 1357	<0.01
Medication			
Diuretics (n, %)	45 (17.7)	29 (59.2)	<0.01
Antiplatelets (n, %)	95 (37.4)	25 (51.0)	NS
Anticoagulants (n, %)	39 (15.4)	22 (44.9)	<0.01
ACE-I/ARBs (n, %)	111 (43.7)	29 (59.2)	NS
β-blockers (n, %)	82 (32.3)	22 (44.9)	NS
Calcium channel blockers (n, %)	94 (37.0)	18 (36.7)	NS
Inotropic agents (n, %)	3 (1.2)	9 (18.4)	<0.01
Statins (n, %)	81 (31.9)	16 (32.7)	NS
Oral hypoglycemic agents (n, %)	45 (17.7)	9 (18.4)	NS
Insulin (n, %)	15 (5.9)	3 (6.1)	NS

Data are presented as mean ± standard deviation or number (percentage)

LDL-cholesterol = low-density lipoprotein cholesterol, HDL-cholesterol = high-density lipoprotein cholesterol, eGFR = estimated glomerular filtration rate, ACE-I = angiotensin converting enzyme inhibitor, ARB = angiotensin-2 receptor blocker

**Fig. 2:**
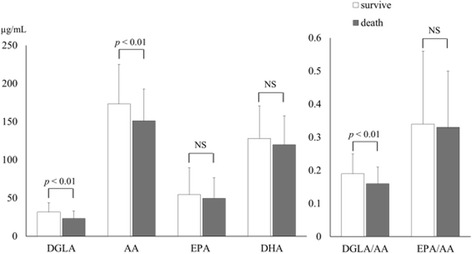
Comparison of polyunsaturated fatty acid levels between the survivor and nonsurvivor groups. Dihomo-gamma-linolenic acid (DGLA) and arachidonic acid (AA) levels and DGLA/AA ratio were significantly lower in the nonsurvivor group than in the survivor group, whereas eicosapentaenoic acid (EPA) and docosahexaenoic acid (DHA) levels and EPA/AA ratio did not differ between groups

As shown in Fig. 2, the DGLA and AA levels were significantly lower in the nonsurvivor group than in the survivor group (DGLA: 23.2 ± 9.8 vs. 31.5 ± 12.0 μg/ml and AA: 151.1 ± 41.6 vs. 173.3 ± 51.6 μg/ml, all *p* < 0.01), although the EPA and DHA levels did not differ between the groups. The DGLA/AA ratio, which indicates the conversion rate of DGLA to AA, was significantly lower in the nonsurvivor group than in the survivor group (0.16 ± 0.05 vs. 0.19 ± 0.06, *p* < 0.01).

In this study population, DGLA was negatively related to age, heart rate, and BNP level and positively related to BMI, systolic blood pressure, diastolic blood pressure, ejection fraction, and eGFR as well as to total cholesterol, LDL-C, HDL-C, TG, total protein, and albumin levels (all *p* < 0.05). AA was negatively related to age, heart rate, and BNP level and positively related to BMI, systolic blood pressure, diastolic blood pressure, and eGFR as well as to total cholesterol, LDL-C, HDL-C, TG, and albumin levels (all *p* < 0.05). EPA was negatively related to BNP level and positively related to age, systolic blood pressure, diastolic blood pressure, and ejection fraction as well as to total cholesterol, LDL-C, and HDL-C levels (all *p* < 0.05). DHA was negatively related to BNP level and positively related to age, systolic blood pressure, diastolic blood pressure, and ejection fraction as well as to total cholesterol, LDL-C, HDL-C, TG, and albumin levels (all *p* < 0.05). DGLA/AA was negatively related to age and BNP level and positively related to BMI and eGFR as well as to total cholesterol, LDL-C, TG, total protein, and albumin levels (all *p* < 0.05). EPA/AA was positively related to age, systolic blood pressure, and diastolic blood pressure (all *p* < 0.05).

**Fig. 3:**
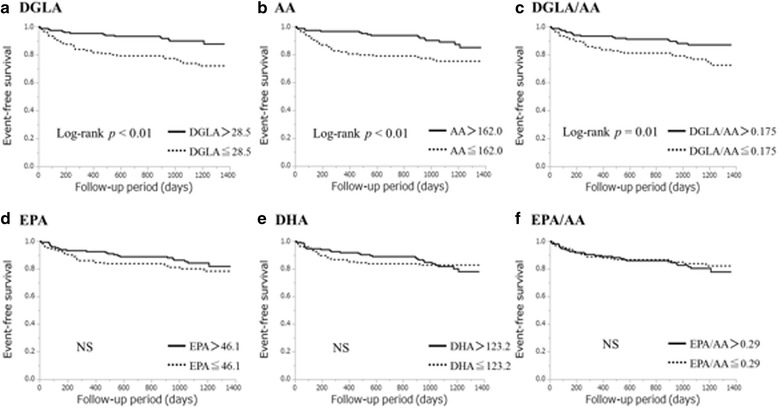
Event-free survival curves for all-cause death in patients with cardiovascular disease. Unadjusted cumulative event rates for the primary endpoint (all-cause death) were estimated using the Kaplan–Meier method and compared between the groups using the log-rank test. We defined cut-offs as the median levels of polyunsaturated fatty acids [dihomo-gamma-linolenic acid (DGLA), 28.5 μg/ml; arachidonic acid (AA), 162.0 μg/ml; eicosapentaenoic acid (EPA), 46.1 μg/ml; docosahexaenoic acid (DHA), 123.2 μg/ml; DGLA/AA, 0.175; EPA/AA, 0.29]

Kaplan–Meier curves were constructed to show the unadjusted event-free rate for all-cause death. We divided the patients into two groups based on the median level of each PUFA (DGLA: 28.5 μg/ml, AA: 162.0 μg/ml, EPA: 46.1 μg/ml, DHA: 123.2 μg/ml, DGLA/AA: 0.175, and EPA/AA: 0.29). The event-free survival rates were higher in the higher DGLA, AA, and DGLA/AA groups than in their lower counterparts (DGLA: *p* < 0.01, AA: *p* < 0.01, and DGLA/AA: *p* = 0.01), although the EPA and DHA levels and the EPA/AA ratio were not associated with prognosis (Fig. 3).

**Fig. 4:**
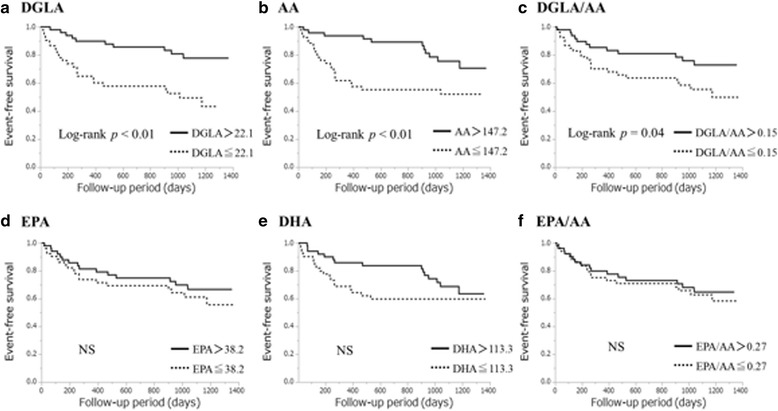
Event-free survival curves for all-cause death in patients with acute decompensated heart failure (ADHF). Unadjusted cumulative event rates for the primary endpoint (all-cause death) were estimated using the Kaplan–Meier method and compared between groups using the log-rank test. We defined cut-offs as the median levels of polyunsaturated fatty acids [ADHF: dihomo-gamma-linolenic acid (DGLA), 22.1 μg/ml; arachidonic acid (AA), 147.2 μg/ml; eicosapentaenoic acid (EPA), 38.2 μg/ml; docosahexaenoic acid (DHA), 113.3 μg/ml; DGLA/AA, 0.15; and EPA/AA, 0.27 and acute coronary syndrome: DGLA, 32.1 μg/ml; AA, 173.0 μg/ml; EPA, 50.7 μg/ml; DHA, 128.8 μg/ml; DGLA/AA, 0.20; and EPA/AA, 0.31]

Furthermore, we investigated the prognosis in patients with ADHF and those with ACS. We divided the patients with ADHF (105 patients) into two groups based on the median level of each PUFA (DGLA: 22.1 μg/ml, AA: 147.2 μg/ml, EPA: 38.2 μg/ml, DHA: 113.3 μg/ml, DGLA/AA: 0.15, and EPA/AA: 0.27). The event-free survival rates were higher in the higher DGLA, AA, and DGLA/AA groups than in their lower counterparts (DGLA: *p* < 0.01, AA: *p* < 0.01, and DGLA/AA: *p* = 0.04; Fig. 4). However, among patients with ACS (129 patients), none of the PUFA levels were associated with prognosis.

Among patients with ADHF, univariate Cox regression analyses showed that age, BMI, dyslipidemia, eGFR, DGLA, AA, DHA, and DGLA/AA, but not EPA and EPA/AA, were associated with long-term mortality (Table 2). After controlling for confounding variables, DGLA and DGLA/AA were associated with long-term mortality (DGLA, 1 μg/ml increase: HR, 0.94; 95% CI, 0.89–0.99; *p* = 0.03 and DGLA/AA, 0.01 μg/ml increase: HR, 0.87; 95% CI, 0.78–0.98; *p* = 0.02), whereas AA, EPA, DHA, and EPA/AA were not associated with long-term mortality (Table 2).

**Table 2:** Univariate and multivariate Cox regression analyses for all-cause death

	Univariate			Multivariate (AA)			Multivariate (DGLA)			Multivariate (DGLA/AA)		
	HR	95% CI	*p*	HR	95% CI	*p*	HR	95% CI	*p*	HR	95% CI	*p*
Age, 1 year increase	1.04	1.01–1.07	<0.01	1.02	0.98–1.06	NS	1.01	0.97–1.05	NS	1.00	0.97–1.04	NS
Male	1.45	0.73–3.02	NS	1.25	0.61–2.71	NS	1.41	0.67–3.12	NS	1.43	0.68–3.14	NS
Body mass index, 1 kg/m^2^ increase	0.90	0.83–0.97	<0.01	0.95	0.87–1.04	NS	0.97	0.88–1.06	NS	0.95	0.87–1.04	NS
Diabetes mellitus	1.24	0.63–2.44	NS	1.29	0.56–3.05	NS	0.96	0.40–2.35	NS	1.11	0.45–2.70	NS
Dyslipidemia	0.38	0.19–0.75	<0.01	0.37	0.15–0.89	0.03	0.47	0.20–1.10	NS	0.37	0.16–0.85	0.02
Hypertension	0.88	0.44–1.83	NS	0.31	0.13–0.73	0.01	0.32	0.13–0.76	0.01	0.34	0.14–0.80	0.01
eGFR, 1 mL/min/1.73m^2^ increase	0.96	0.94–0.98	<0.01	0.94	0.91–0.97	<0.01	0.94	0.91–0.97	<0.01	0.94	0.91–0.96	<0.01
Atrial fibrillation	1.05	0.53–2.07	NS	1.22	0.51–2.80	NS	1.07	0.45–2.43	NS	1.25	0.54–2.80	NS
Ejection fraction, 1% increase	1.00	0.97–1.02	NS	0.98	0.96–1.01	NS	0.99	0.97–1.01	NS	0.99	0.97–1.01	NS
AA, 1 μg/mL increase	0.99	0.98–1.00	0.01	1.00	0.99–1.01	NS		-			-	
DGLA, 1 μg/mL increase	0.92	0.88–0.96	<0.01		-		0.94	0.89–0.99	0.03		-	
DGLA/AA, 0.01 increase	0.92	0.85–0.98	0.01		-			-		0.87	0.78–0.98	0.02

*eGFR* Estimated glomerular filtration rate, *AA* Arachidonic acid, *DGLA* Dihomo-gamma-linolenic acid

## Discussion

The present study demonstrated that low serum levels of omega-6 PUFAs in patients with acute cardiovascular disease could predict poor long-term prognosis, but omega-3 PUFA levels were not associated with prognosis. Particularly in patients with ADHF, lower serum levels of DGLA and a lower DGLA/AA ratio could predict poor long-term prognosis, whereas in patients with ACS, none of the PUFA levels were associated with prognosis.

A recent study reported that low levels of omega-6 PUFAs (the sum of AA and DGLA levels) was associated with poor prognosis in patients with ADHF [26]. However, it is well known that AA and DGLA have opposing effects, partly through their metabolites. PUFA metabolites form precursors to prostaglandins, thromboxanes, and leukotrienes [27, 28]. AA is converted to series 2 prostaglandins and series 4 leukotrienes, which have pro-inflammatory potential and the ability to induce platelet aggregation and vasoconstriction [29, 30]. Although AA is associated with inflammation, atherosclerosis, and a hypercoagulable state, DGLA is converted to series 1 prostaglandins and series 3 leukotrienes, which have anti-inflammatory effects [5, 22, 31–33]. These metabolites also inhibit the effects of AA-derived metabolites. Moreover, a high DGLA/AA ratio indicates a decrease in the endogenous production of AA by delta-5 desaturase. This may explain the independent association of high DGLA levels and a high DGLA/AA ratio, but not AA levels, with better prognosis in this study population.

Omega-3 PUFA derivatives play a significant role in the process of blood coagulation and inflammation [34, 35]. Many studies have reported that omega-3 PUFAs help lower the risk of cardiovascular diseases. However, in the present study, omega-3 PUFAs were not associated with long-term mortality. Therefore, further studies are warranted to clarify the effects of omega-3 PUFAs, unlike omega-6 PUFAs, in the treatment of acute cardiovascular diseases.

### Limitations

The present study had several limitations. This study was performed in a single institution, and the study population was small. Moreover, the study duration was short; therefore, it may be difficult to generalize the results. Studies with a larger sample size and longer duration are needed to assess the association between PUFAs and prognosis in patients with cardiovascular diseases.

## Conclusion

Low serum DGLA levels and a low DGLA/AA ratio predict long-term mortality in patients with acute cardiovascular diseases, particularly those with ADHF, suggesting that low serum levels of omega-6 PUFAs may be a useful predictor and potential therapeutic target in these patients.

## Additional file

Additional file 1: Serum fatty acids profiles of survivors and nonsurvivors. (XLSX 32 kb)

## Abbreviations

AA: Arachidonic acid
ACS: Acute coronary syndrome
ADHF: Acute decompensated heart failure
BMI: Body mass index
CIs: Confidence intervals
DGLA: Dihomo-gamma-linolenic acid
DHA: Docosahexaenoic acid
eGFR: Estimated glomerular filtration rate
EPA: Eicosapentaenoic acid
HDL-C: High-density lipoprotein cholesterol
HRs: Hazard ratios
LDL-C: Low-density lipoprotein cholesterol
PUFA: Polyunsaturated fatty acid
TG: Triglyceride
